# Education Research: Quality and Validity Evidence for a National In-Training Examination for Epilepsy Fellows

**DOI:** 10.1212/NE9.0000000000200090

**Published:** 2023-10-26

**Authors:** Jeremy Moeller, Ernesto Gonzalez-Giraldo, France W. Fung, Emily L. Johnson, Ammar Kheder, Jane MacLean, Emily L. McGinnis, Wolfgang G. Muhlhofer, Joel M. Oster, Sarah Schmitt, P. Emanuela Voinescu, Lily C. Wong-Kisiel, Kandice J. Kidd, Amy Kephart, Fred A. Lado, Sudha Kilaru Kessler

**Affiliations:** From the Department of Neurology (J. Moeller), Yale School of Medicine, New Haven, CT; Departments of Neurology and Pediatrics (E.G.-G.), University of California, San Francisco; Departments of Neurology and Pediatrics (F.W.F., S.K.K.), Children's Hospital of Philadelphia and University of Pennsylvania Perelman School of Medicine, Philadelphia; Department of Neurology (E.L.J.), Johns Hopkins School of Medicine, Baltimore, MD; Department of Neurology and Pediatric Institute (A. Kheder), Emory University School of Medicine, Atlanta, GA; Department of Pediatric Neurology (J. MacLean), Sutter Medical Foundation, Mountain View, CA; Neurology Department (E.L.M.), Kaiser Permanente Los Angeles Medical Center, CA; University of Washington Regional Epilepsy Center (W.G.M.), University of Washington, Seattle; Department of Neurology (J.M.O.), Tufts University, Boston, MA; Department of Neurology (S.S.), Medical University of South Carolina, Charleston; Division of Epilepsy (P.E.V.), Department of Neurology, Brigham and Womens Hospital, Harvard Medical School, Boston, MA; Department of Neurology (L.C.W.-K.), Mayo Clinic College of Medicine, Rochester, MN; American Epilepsy Society (K.J.K., A. Kephart), Chicago, IL; and Department of Neurology (F.A.L.), Zucker School of Medicine at Hofstra-Northwell, Hempstead, NY.

## Abstract

**Background and Objectives:**

Epilepsy education has been transformed over the past 2 decades, leading to a need for structured formative assessment tools. The American Epilepsy Society developed the Epilepsy Fellowship In-Training Examination (EpiFITE) to provide high-quality formative assessment for fellows, to stimulate program improvement, and to guide future learning and teaching. The aim of this study was to explore validity evidence for the EpiFITE in meeting these goals.

**Methods:**

Validity evidence was sought from multiple sources. The content of the examination was linked to the American Board of Psychiatry and Neurology blueprint for initial certification in epilepsy, and items were developed by trained experts. Internal structure was studied using internal consistency and item analysis. Surveys of fellows and fellowship directors focused on the examination experience (response process) and how results influenced fellow assessment, future learning, and program improvement (relationship to other variables and consequences).

**Results:**

The EpiFITE was first administered in 2020, with 172 examinees from 67 programs. By 2022 (year 3), the EpiFITE was completed by 195 epilepsy fellows from 77 programs. The overall mean score of the examination was stable from year to year, and the committee predicted the difficulty of individual items with a high degree of accuracy. The examination had high internal consistency (Cronbach α 0.76–0.81). The median item discrimination index ranged from 0.17 in 2020 to 0.21 in 2022. Discrimination indices were lower (mean ≤0.10) for items that were either very easy or very difficult and significantly higher (mean >0.20) for other items. Program directors and epilepsy fellows agreed the examination questions were appropriate and agreed that the EpiFITE helped them identify areas for self-directed learning. Program directors also found the examination helpful in identifying areas of strength and areas for improvement within their programs.

**Discussion:**

There are several sources of evidence of the quality and validity of the EpiFITE. By exploring this validity evidence, we have identified several best practices in the development and evaluation of a subspecialty examination, and this experience could be helpful for developers of in-training examinations in other subspecialties.

## Introduction

Epilepsy training has undergone a transformation over the past 20 years. The number of Accreditation Council for Graduate Medical Education (ACGME)–accredited epilepsy fellowships has increased from 11 programs with 12 total fellows in 2014–2015 to 94 programs with 154 total fellows in 2021–2022.^1,2^ This growth has spurred a need for formative assessment materials to ensure that fellows are reaching the goals of subspecialty training. In the past, certification examinations for epileptologists focused on clinical neurophysiology and electroencephalography and did not assess many other evolving elements of specialized epilepsy care.^3^ In addition, there is an active discussion in the neurology community about redefining clinical neurophysiology programs to focus on training in a broad range of specialized techniques in inpatient and outpatient neurology, and there is likely to be increasing divergence between epilepsy and clinical neurophysiology training.^4^

In nearly every aspect of epilepsy care, there has been enormous growth in the knowledge and skills required of an epilepsy specialist. In the past 30 years, the number of approved antiseizure medications has increased 3-fold, and many of the newest medications have complicated pharmacologic considerations.^5^ Surgical options for epilepsy now include not only resection and disconnection procedures but also multiple neurostimulation options, including responsive neurostimulation and deep brain stimulation with multiple potential targets.^6^ There have been significant advances in our understanding of how to treat epilepsy in childhood,^7^ in pregnancy,^8^ and in patients with systemic disease, especially genetic and autoimmune disorders.^9,10^ There are many more treatment options for status epilepticus and an evolving understanding of how to identify those individuals most likely to benefit from aggressive treatment.^11^ Beyond the biomedical aspects of epilepsy care, epilepsy specialists require expertise in the psychosocial aspects of care, including an increasingly sophisticated understanding of anxiety, mood disorders, and psychogenic nonepileptic attacks.^12^ In addition, epilepsy specialists must recognize the impact of sex, socioeconomic status, race, and other sources of health disparities on epilepsy treatment and outcome.^13^

In response to the growth in the breadth of epilepsy-specific knowledge required by trainees in accredited epilepsy programs, the American Epilepsy Society (AES) created the Epilepsy Fellowship In-Training Examination (EpiFITE) to provide a standardized assessment tool focusing not only on the competencies of medical knowledge and patient care but also on other competencies essential to subspecialty training in epilepsy.

In the process of developing this examination, we aimed to ensure that the results of the examination were an accurate reflection of expertise in epilepsy care. In multiple choice examinations, evidence of validity can be found using several sources.^14^ The content of the examination depends on a well-chosen blueprint, expert item writers, and a process for ensuring appropriate quality and difficulty of the items themselves.^15^ The internal structure of the examination can be evaluated using multiple statistical approaches, including measurements of internal consistency of the entire examination and analysis of individual items.^16^ Response process is the degree to which the intended purpose of the test aligns with the thought process and actions of the test-takers, and evidence can be found through learner feedback and by ensuring that the test is administered and scored using a structured and secure approach.^17^ Finally, validity evidence can be demonstrated by showing a relationship between examination results and other variables that are aligned with learning objectives, such as performance in other domains or future assessments, and—perhaps most importantly for assessments in medical education—provision of high-quality patient care.

While the EpiFITE is a formative assessment tool with much lower stakes than certification examinations, we aimed to ensure that there was strong validity evidence so that the EpiFITE is of greatest value to all stakeholders. Other studies have shown that results of in-training examinations can have strong correlation with performance on certification examinations,^18,19^ so we also aimed to ensure that the EpiFITE would help fellows focus their preparation for the American Board of Psychiatry and Neurology (ABPN) initial certification examination in epilepsy. For fellowship program directors, we aimed to provide detailed metrics of each fellow's knowledge base providing support for the development of individualized learning plans. We also aimed to provide program-level data and year-to-year standardized assessment data with comparisons with national results for the purposes of program evaluation and improvement. For the AES and the community of epilepsy practitioners, our goal was to promote high-quality epilepsy education and training, thus enhancing care of patients with epilepsy.

In this study, we describe the steps in developing the EpiFITE, including an examination of multiple sources of validity evidence so that we can ensure that this examination meets a growing need for formative assessment in subspecialty epilepsy education. We also aimed to apply our findings to recommendations for other neurology subspecialty organizations who are considering the development of their own in-training examinations.

## Methods

### Standard Protocol Approvals, Registrations, and Patient Consents

This work was determined to be exempt from annual institutional review board review by the Yale Human Research Protection Program (IRB Protocol ID 2000033656).

### Content

Committee members were recruited with 2 main qualifications: expertise in epilepsy and experience in educating epilepsy fellows. We sought to ensure that there was a diverse committee membership, with a balance of pediatric and adult neurologists, broad institutional representation throughout the United States, private and academic practices, and a range of specific expertise in both medical and surgical treatment of epilepsy. Committee members were all board certified in epilepsy, had experience in graduate medical education, and had between 2 and 30 years of postfellowship clinical experience. All but 2 committee members were located at academic medical centers.

The committee was assigned the task of developing a 110-item examination with a range of questions that adhered to the ABPN Certification Examination Blueprint (Table 1).^20^ Additional sources of guidance on examination content included the ACGME Epilepsy Milestones and the ACGME Common Program Requirements for Epilepsy Fellowship.^21,22^ Overall, the proportion of topics matched very closely with the ABPN blueprint, although the committee opted to include slightly more questions focusing on pathophysiology and pathology because these were thought to be less well covered in routine fellowship training and therefore could be useful for formative feedback for fellows and program directors. Subtopics generally matched closely to the topics outlined in the ABPN blueprint, and the overall format was approved by the committee before the first year. No major changes in the EpiFITE blueprint were made in subsequent years. The 110-item examination was chosen as approximately half of the number of items in the ABPN certification examination. A shorter examination was chosen to ensure that the EpiFITE could be completed in a half day, without excessive disruption of training or patient care.

**Table 1:**
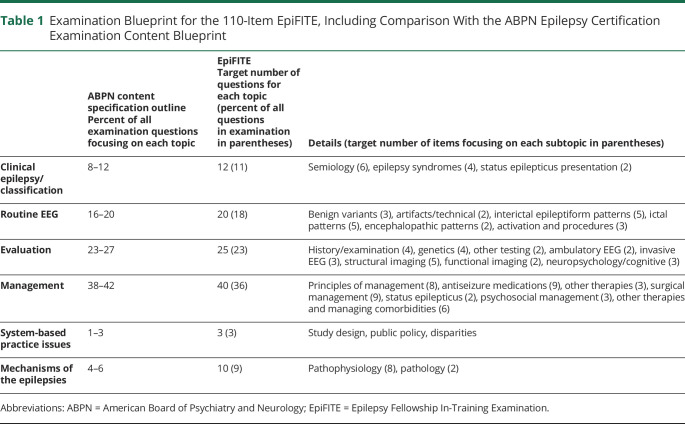
Examination Blueprint for the 110-Item EpiFITE, Including Comparison With the ABPN Epilepsy Certification Examination Content Blueprint

	ABPN content specification outline Percent of all examination questions focusing on each topic	EpiFITE Target number of questions for each topic (percent of all questions in examination in parentheses)	Details (target number of items focusing on each subtopic in parentheses)
Clinical epilepsy/classification	8–12	12 (11)	Semiology (6), epilepsy syndromes (4), status epilepticus presentation (2)
Routine EEG	16–20	20 (18)	Benign variants (3), artifacts/technical (2), interictal epileptiform patterns (5), ictal patterns (5), encephalopathic patterns (2), activation and procedures (3)
Evaluation	23–27	25 (23)	History/examination (4), genetics (4), other testing (2), ambulatory EEG (2), invasive EEG (3), structural imaging (5), functional imaging (2), neuropsychology/cognitive (3)
Management	38–42	40 (36)	Principles of management (8), antiseizure medications (9), other therapies (3), surgical management (9), status epilepticus (2), psychosocial management (3), other therapies and managing comorbidities (6)
System-based practice issues	1–3	3 (3)	Study design, public policy, disparities
Mechanisms of the epilepsies	4–6	10 (9)	Pathophysiology (8), pathology (2)

Abbreviations: ABPN = American Board of Psychiatry and Neurology; EpiFITE = Epilepsy Fellowship In-Training Examination.

For the first year of the examination, an independent consultant was engaged to provide process expertise in examination development, item writing, and editing. Committee members underwent a faculty development session to gain skills in developing high-quality multiple-choice items, and then each faculty member was assigned to create 6–8 original questions and to independently review and edit 10–20 items. Faculty members generated a question stem, a single correct answer with 4 distractors, a short topic discussion, and a list of 1–3 references. Emphasis was placed on avoiding low-quality formats such as multiple true-false questions (“which of these answers is true?”), negative question stems (“all of these are true EXCEPT…”), and compound answers.^23^ A major emphasis was placed on testing content related to the practice of epilepsy care and avoiding questions that focused on the recall of less clinically relevant facts. To that end, many items included clinical images, such as diagnostic imaging (MRI, PET, fMRI, etc.) and screenshots or brief epochs (2–3 pages) of EEG examples. Each item was reviewed by the independent consultant, with a focus on item quality, and by the committee chair for item quality and content. Once the initial items were drafted, the group met at the AES Annual Meeting in December 2019 to review several items as a group and to continue the process of developing a shared mental model of item quality and difficulty level. At an additional full-day in-person meeting in February 2020, all remaining items were reviewed and edited by the entire group. A total of 130 items were created, and from these, 110 items were selected by the chair for the final version of the 2020 examination.

The committee had discussions about the balance of item difficulty in the context of the planned use of individual scores.^17^ We aimed to develop an examination with a wide range of item difficulty, each with a different purpose.^16^ Low-difficulty items could ensure that all fellows were demonstrating “mastery” of core concepts. Medium-difficulty items could allow a high degree of discrimination among fellows, which could provide valuable predictive data for both fellows and programs in preparation for the high-stakes certification examination. High-difficulty items could challenge fellows, educating them about the vast range of knowledge, skills, and attitudes that one can engage in epilepsy care. Because the EpiFITE was an educational tool, we did not set a specific target or cutoff score.

The intention was to develop a “bank” of items over several years, so that for each edition of the examination, there would be some items that were reused from prior editions and some new items. For each of 2021 and 2022, a rotating group of committee members developed 60–70 new items, which were reviewed according to the same process as the 2020 edition, except that all committee meetings were virtual because of the coronavirus disease 2019 (COVID-19) pandemic. New items were supplemented by items from prior editions of the examination: the prior items were selected so that the entire examination remained balanced according to the ABPN blueprint, and new items often focused on emerging concepts so that the content remained up to date. The cochairs also ensured that any reused items were reviewed and updated if necessary so that items were balanced according to difficulty and that poorly performing items were excluded from reuse.

In an additional step to ensure the content of the examination was appropriately difficult, we studied the relationship between predicted difficulty and actual difficulty of each item. The committee assigned a consensus score for predicted difficulty on a 4-point scale to each new item. Using an adaptation of the modified Angoff approach,^24^ committee members were instructed to assign a score of 1 if the average epilepsy fellow could be expected to answer the questions correctly 75%–100% of the time, 2 if the average fellow would be correct 50%–75% of the time, 3 for 25%–50% of the time, and 4 if the average fellow would be correct <25% of the time. We then evaluated whether our predictions matched actual test performance by comparing our predicted difficulty scores with the actual item difficulty index. We included only the first use of each item, when we did not have any additional item analysis to influence our predictions.

### Internal Structure

The overall internal consistency of the examination was determined using Cronbach α. Item analysis included difficulty index and discrimination index. These values were not available for analysis between 2020 and 2021, but for the 2022 version of EpiFITE, we reviewed difficulty and discrimination index for any questions that were repeated from prior years and excluded items with a negative discrimination index. We opted to use discrimination index for our initial analysis because this was the metric provided through the learning management system. To ensure thorough item analysis for the purposes of this study, we also independently calculated the point biserial correlation coefficient for each item and compared this with the discrimination index in our final analysis.

We adapted a previously described framework for categorization of item difficulty to investigate the relationship between item difficulty and discrimination index and to compare the distribution of item difficulty for all 3 years of the examination.^16^ In this framework, items were categorized into 5 different levels based on difficulty index: easiest (>90% examinees examined correctly), easy (75%–90% correct), medium (45%–74% correct), difficult (25%–44% correct), and very difficult (<25% correct). We also examined item difficulty and discrimination index as a function of the 6 main topic categories outlined in Table 1. For the topic category analyses, we included only the first use of each item, so that repeated items were not counted more than once.

### Response Process

We paid careful attention to the administration of the examination to ensure that examinees and program directors understood the purpose of the EpiFITE and were able to engage with the examination without major limitations. Preceding the initial examination in 2020, the AES advertised to fellowship program directors by email and at its annual meeting. Registration instructions discouraged programs from allowing fellows to take the examination a second time. Registration facilitated per-fellow payment and access to the web-based platform used for test administration. Detailed instructions were provided to program contacts (typically the program director and/or program coordinator). The testing period occurred over 1 full week that spanned a weekend to allow for programs to choose a date that worked with their scheduling constraints. The time allotted for the 110-item examination was 180 minutes. An AES staff member was on-call for troubleshooting during the days of test administration. A suggested proctoring plan was provided to programs, but because the first test took place in March 2020 (just after the national COVID-19 pandemic lockdown), programs were given discretion on testing location and how to implement local proctoring. On completion of the test, each fellow was provided with an overall percent correct score. Because of planned reuse of items, answers and explanations, with medical literature references, were provided only for the items each fellow answered incorrectly, but not for the entire examination. Data that allowed programs to compare each fellow's performance with the national cohort of fellows and programs were provided 2 weeks after examination administration, allowing time to ensure accuracy of the data. A postexamination survey was sent to fellows within 1 month of examination completion (in 2020 and 2022), and this survey included questions about the ease of use of the examination platform, appropriate distribution of question topics, and the utility of the examination in identifying learning gaps.

### Relationship to Other Variables and Consequences

To determine relationship to other variables, we analyzed several items on the postexamination surveys of fellows and program directors. Fellows were asked whether the EpiFITE helped guide preparation for the ABPN certification examination, and fellowship directors were asked whether the EpiFITE helped identify areas of strength and areas for improvement, informed medical knowledge milestones, and helped guide preparation for the certification examination.

### Statistical Analysis

Item analysis (difficulty index, discrimination index, Cronbach α) was provided by the learning management system (Oasis LMS, Chicago, IL), and all other statistical analyses were performed using SPSS Statistics (version 28.0.0.0). Mean values were compared with 1-way analysis of variance tests, medians were compared using the independent samples median test, and correlations between performance on test items from year to year were compared using the 2-tailed Pearson correlation. Correlation between predicted difficulty and actual difficulty index was performed by calculating the 1-tailed Spearman correlation.

### Data Availability

Anonymized data not published within this article will be made available by request from any qualified investigator.

## Results

### Examination Cohort, Content, and Overall Results

In the first year of the examination, 172 fellows from 67 programs completed the examination, and by 2022, there were 195 fellows from 77 different programs (Table 2). Specific data were not available regarding the type of fellowship program (epilepsy vs clinical neurophysiology, accredited vs nonaccredited, adult vs pediatric) or year of training. However, considering that there were only 154 epilepsy trainees in accredited epilepsy programs in 2022, it is likely that at least some trainees were in other training programs. Overall, the examination took approximately 2 hours to complete and examinees took significantly longer in 2021 compared with that in the other 2 years. The overall mean score was stable at approximately 66% for all 3 years of the examination, but subcategory scores varied from year to year (Table 2).

**Table 2:**
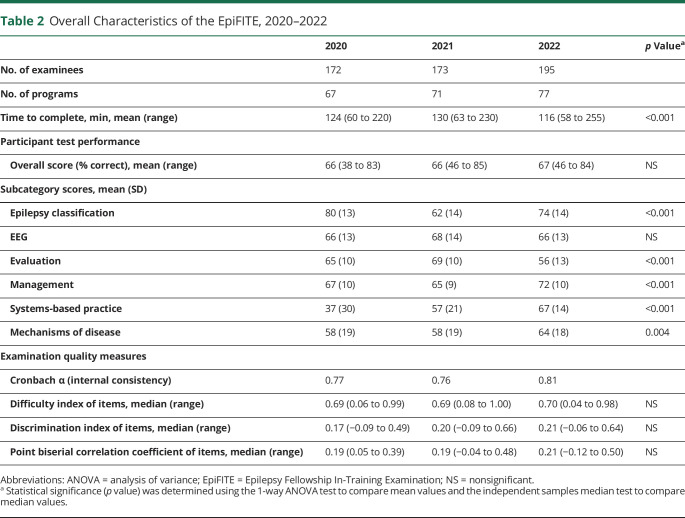
Overall Characteristics of the EpiFITE, 2020–2022

	2020	2021	2022	*p* Value^a^
No. of examinees	172	173	195	
No. of programs	67	71	77	
Time to complete, min, mean (range)	124 (60 to 220)	130 (63 to 230)	116 (58 to 255)	<0.001
Participant test performance				
Overall score (% correct), mean (range)	66 (38 to 83)	66 (46 to 85)	67 (46 to 84)	NS
Subcategory scores, mean (SD)				
Epilepsy classification	80 (13)	62 (14)	74 (14)	<0.001
EEG	66 (13)	68 (14)	66 (13)	NS
Evaluation	65 (10)	69 (10)	56 (13)	<0.001
Management	67 (10)	65 (9)	72 (10)	<0.001
Systems-based practice	37 (30)	57 (21)	67 (14)	<0.001
Mechanisms of disease	58 (19)	58 (19)	64 (18)	0.004
Examination quality measures				
Cronbach α (internal consistency)	0.77	0.76	0.81	
Difficulty index of items, median (range)	0.69 (0.06 to 0.99)	0.69 (0.08 to 1.00)	0.70 (0.04 to 0.98)	NS
Discrimination index of items, median (range)	0.17 (−0.09 to 0.49)	0.20 (−0.09 to 0.66)	0.21 (−0.06 to 0.64)	NS
Point biserial correlation coefficient of items, median (range)	0.19 (0.05 to 0.39)	0.19 (−0.04 to 0.48)	0.21 (−0.12 to 0.50)	NS

Abbreviations: ANOVA = analysis of variance; EpiFITE = Epilepsy Fellowship In-Training Examination; NS = nonsignificant.

a Statistical significance (*p* value) was determined using the 1-way ANOVA test to compare mean values and the independent samples median test to compare median values.

**Figure 1:**
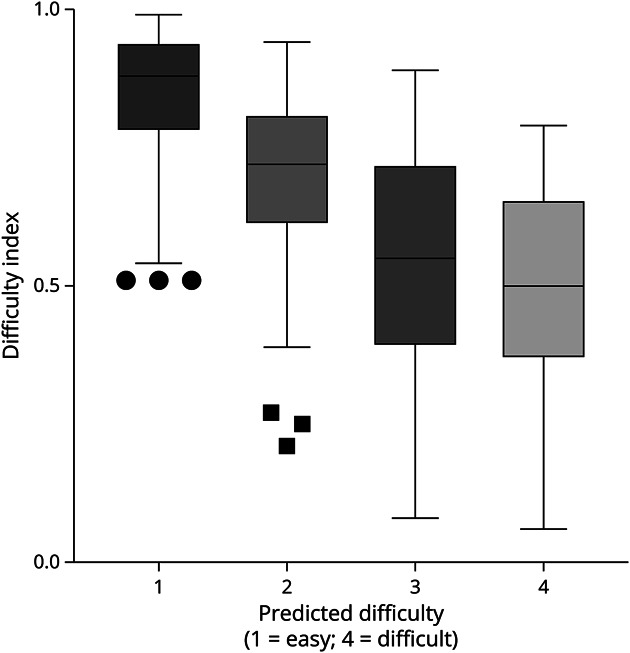
Difficulty Index (on First Administration) as a Function of Predicted Difficulty for All Original Items, From 2020 to 2022 (N = 217 Original Items for Which Difficulty Was Predicted) Item difficulty ranges from 0 to 1.0 and is calculated as the proportion of examinees who answered a question correctly, so that a lower score represents a more difficult item. Predicted difficulty was estimated by committee consensus as a range from 1 = easiest items (the average candidate would answer these questions correctly 75%–100% of the time) to 4 = most difficult items (the average candidate would be predicted to answer these questions less than 25% of the time). Correlation between these items was statistically significant (Spearman correlation coefficient 0.612; *p* < 0.001).

For the 2021 examination, 44 items from the 2020 examination were reused, and 66 new items were included. In 2022, 39 items from 2020 and 22 items from 2021 were reused, and there were 49 new items. For items that were repeated, the item difficulty scores remained highly consistent from year to year (Pearson correlation 0.91 to 0.94, *p* < 0.001 for all year-to-year comparisons). The predicted difficulty of faculty members matched well with the actual difficulty of items as determined by difficulty index, as outlined in Figure 1.

### Internal Structure

**Figure 2:**
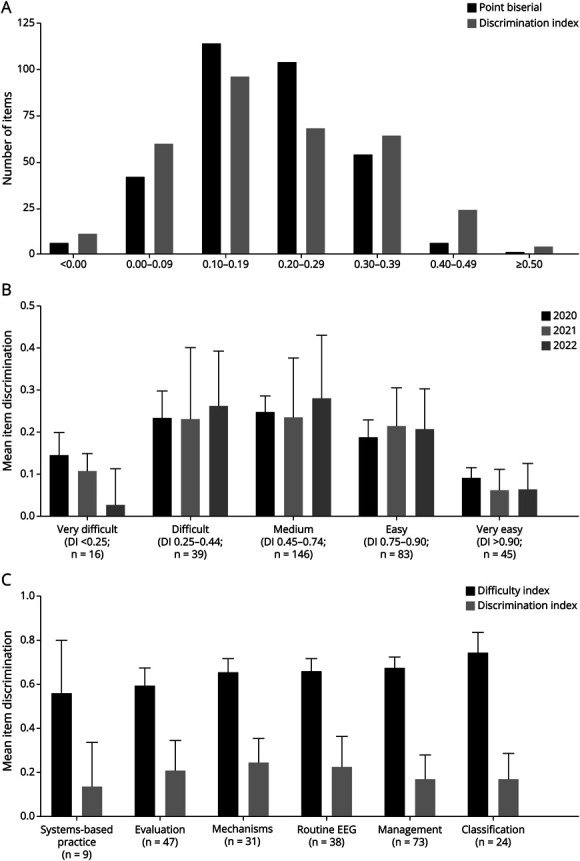
Summary of Item Analysis Metrics for the EpiFITE Examination, 2020–2022 (A) Histogram of the distribution of both point biserial correlation coefficient and DI for all items from 2020 to 2022. For both measures, approximately 50% of the items had a value of at least 0.20. Only 2%–3% of items had a value of less than 0, suggesting invalid items. (B) Relationship between item difficulty (difficulty index ranges noted in parentheses) and mean DI for all items in each year's version of the EpiFITE, showing higher discrimination for moderate-difficulty items and lower discriminatory value for very easy and very difficult items (*p* < 0.001 by ANOVA). (C) DI and difficulty index for all original items (in the first version of the examination in which they appeared) by topic subcategory. The difference in difficulty index between categories was not significant (*p* = 0.08), but there was a significant difference between the mean discrimination indices of items in each subcategory, with categories with overall moderate difficulty showing higher mean levels of discrimination compared with more difficult or easier categories (*p* = 0.04). ANOVA = analysis of variance; DI = discrimination index; EpiFITE = Epilepsy Fellowship In-Training Examination.

The Cronbach α ranged from 0.76 to 0.81 across all 3 years of examination administration, suggesting good internal consistency and reliability of the examination (Table 2). The median discrimination index ranged from 0.17 in 2020 to 0.21 in 2022 (*p* = nonsignificant [NS]), and the median point biserial coefficient ranged from 0.19 in 2020 to 0.21 in 2022 (*p* = NS). There was a similar range of item discrimination using the discrimination index and point biserial correlation coefficient (Figure 2A). Overall, approximately half of the items had a discrimination index and point biserial coefficient of at least 0.20. The easiest and most difficult items generally had a lower mean discrimination index, while items with moderate difficulty had significantly higher mean discrimination indices (Figure 2B). Over the 3 years, there was no significant difference in difficulty index between the subcategories, although there was a trend for items that focused on systems-based practice to be the most difficult, while items focusing on classification trended as the easiest (Figure 2C). Items in the easiest and most difficult categories had the lowest discrimination, while items in the moderately difficult categories had the highest discrimination (Figure 2C).

### Response Process, Relationship to Other Variables, and Consequences

Fellowship director and fellow surveys were conducted in 2020 and 2022 and response rates for fellows and faculty were 34% and 36%, respectively, in 2020 and 44% and 58%, respectively, in 2022 (Table 3). Consistent responses were seen in both iterations of the survey. Regarding response process, most of the fellows agreed or strongly agreed that the question topics were appropriately distributed and that the examination platform was easy to use. In terms of relationships to other variables, most of the fellowship directors said that they would use the EpiFITE to inform knowledge milestones and thought that the EpiFITE helped identify areas of strength and areas for improvement. Regarding consequences, nearly all program directors and 86%–93% of fellows agreed that this examination would be a useful method of preparing for the additional certification examinations through the ABPN. Large majorities of both groups agreed that the examination was a valuable resource and should be recommended for future fellows and used in future years.

**Table 3:**
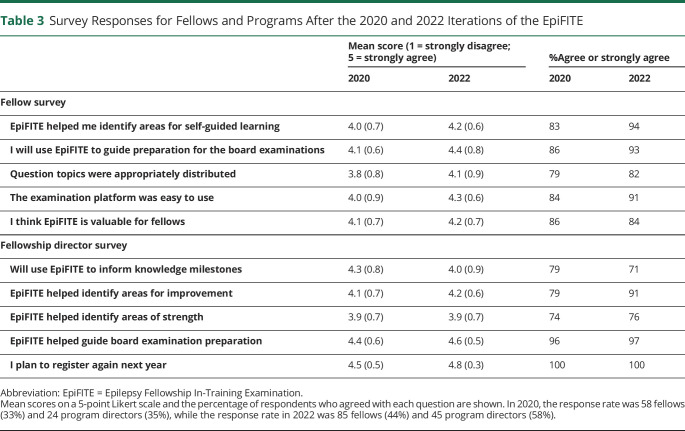
Survey Responses for Fellows and Programs After the 2020 and 2022 Iterations of the EpiFITE

	Mean score (1 = strongly disagree; 5 = strongly agree)		%Agree or strongly agree	
	2020	2022	2020	2022
Fellow survey				
EpiFITE helped me identify areas for self-guided learning	4.0 (0.7)	4.2 (0.6)	83	94
I will use EpiFITE to guide preparation for the board examinations	4.1 (0.6)	4.4 (0.8)	86	93
Question topics were appropriately distributed	3.8 (0.8)	4.1 (0.9)	79	82
The examination platform was easy to use	4.0 (0.9)	4.3 (0.6)	84	91
I think EpiFITE is valuable for fellows	4.1 (0.7)	4.2 (0.7)	86	84
Fellowship director survey				
Will use EpiFITE to inform knowledge milestones	4.3 (0.8)	4.0 (0.9)	79	71
EpiFITE helped identify areas for improvement	4.1 (0.7)	4.2 (0.6)	79	91
EpiFITE helped identify areas of strength	3.9 (0.7)	3.9 (0.7)	74	76
EpiFITE helped guide board examination preparation	4.4 (0.6)	4.6 (0.5)	96	97
I plan to register again next year	4.5 (0.5)	4.8 (0.3)	100	100

Abbreviation: EpiFITE = Epilepsy Fellowship In-Training Examination.

Mean scores on a 5-point Likert scale and the percentage of respondents who agreed with each question are shown. In 2020, the response rate was 58 fellows (33%) and 24 program directors (35%), while the response rate in 2022 was 85 fellows (44%) and 45 program directors (58%).

## Discussion

Using a framework of a clearly stated set of objectives and careful attention to examination quality and validity, we identified strong evidence that the EpiFITE was successful in achieving its aims. Careful attention was paid to examination content to ensure that there was a clear focus on the level of knowledge and clinical care that would be expected of a clinical epilepsy fellow. There was consistent performance from year to year, and committee members were able to accurately predict the difficulty of individual items. A wide mixture of item difficulty levels ensured that the examination had discriminatory value, could demonstrate mastery, and could challenge fellows to expand their knowledge. The examination had a high degree of internal consistency, suggesting a strong overall internal structure. Finally, both fellows and program directors agreed that the examination could be used to inform self-directed learning, high-stakes examination preparation, and program improvement.

One potential weakness of the EpiFITE was the relatively low discrimination indices for some individual items. As expected, the discrimination indices were highest for items of moderate difficulty because of a higher expected variance in individual performance.^16^ Very easy and very difficult items typically have lower discrimination indices (because either very few or almost all examinees get these questions correct), and this was the case with the EpiFITE.^16^ Except for these items, the mean overall discrimination index was above 0.20. In addition, there was a trend toward improvement of the mean discrimination index for moderate difficulty items in 2022, possibly based on our attempts to exclude items from prior examinations with poor performance characteristics. We intended to produce a test with a broad range of difficulty levels to ensure that some items would test core concepts, others would be helpful in discerning the highest performers, and there would be a small number of “challenge” items to prompt further self-directed learning. That said, the most difficult items (“very difficult” from Figure 1B) are not highly discriminating and may be of limited educational value. This represents a very small number of items (16 items over the 3 years of this study), and excluding these few items from reuse in future examinations could improve internal consistency without sacrificing the rigor of the examination. Of note, there was no statistical difference in item difficulty based on topic subcategory. This suggests that the variability in difficulty of items was not clearly related to specific content and is possibly more a function of item construction. For example, it may be possible to make an “easy” question that focuses on the most difficult content and vice versa.

An additional potential source of the relatively low levels of discrimination for individual items may relate to variability in the structure of postgraduate epilepsy training. Some trainees choose to train in epilepsy programs, some choose clinical neurophysiology programs, and some choose programs with both subspecialties. In addition, there are programs that offer non-ACGME accredited additional years of training. Each trainee could have specific areas of strength and weakness that are related more to the heterogenous nature of training programs than to the individual strength of examinees. In addition, it may be hard to discriminate based on year of training because of a typical emphasis on research and epilepsy surgical evaluations during a second year of training. While these skills are important for the longitudinal development of a fellow's career, they represent only a small proportion of the ABPN epilepsy assessment evaluation blueprints and thus a small proportion of the items on this examination. Another reason for poor discrimination could be the variability among programs between pediatrics-focused training and adult-focused training. Unfortunately, with our existing data, it was not possible to distinguish fully between adult and pediatric epilepsy fellows or programs to determine the magnitude of this effect. The validity of the examination could also be negatively affected by fellows taking the examination twice (e.g., during their first and second years of fellowship), although this practice was discouraged. It is likely that this had a relatively minor impact on validity, considering that for items that were repeated, the difficulty index remained highly consistent from year to year.

We acknowledge that a major limitation of the EpiFITE, as with all multiple-choice format standardized in-training assessments, is that the primary focus is assessment of medical knowledge and a trainee's ability to apply that knowledge to realistic but highly structured clinical vignettes. A competent epilepsy specialist must also demonstrate achievement of many competencies and subcompetencies that cannot be assessed with any written examination. For example, although the EpiFITE included many items that contained screenshots of EEG, a trainee's ability to correctly interpret the relevant findings on these images does not necessarily indicate competence in EEG interpretation. Future versions of this and other examinations could include videos and advanced EEG elements (e.g., scrolling through multiple screens, changes in filter and montage settings, etc.) to assess more advanced skills. The quality and validity of such elements could be evaluated using a similar process to the one we have described.

In-training examinations have a well-established tradition in neurology and provide valuable feedback to both trainees and program directors that cannot be gained through other methods. Since 1988, the American Academy of Neurology has offered the Residency In-Service Training Examination (RITE) to neurology trainees, and 2 publications have shown a high degree of correlation between RITE examination performance and eventual ABPN initial certification examinations.^19,25^ Correlation between in-training examination performance and success on the initial certification examination has also been shown in psychiatry, nephrology, and hematology/oncology.^18,26,27^ In the case of hematology/oncology, performance on the in-service examination was more closely correlated with success on the initial certification examination than the ratings of medical knowledge determined by program directors, suggesting that national in-service examinations can provide assessment data that are not easily obtained at a local level.^27^ This aligns well with our survey data, in which most of both fellows and program directors responded that the EpiFITE allowed them to identify areas for improvement and guidance for board preparation. While we have not yet evaluated whether results on the EpiFITE can predict performance on the initial certification examination in epilepsy, this could provide further validation data for our examination.

The number of ACGME-accredited neurologic subspecialties with training programs has grown, and there is an even greater number of neurologic subspecialties that offer subspecialty certification examinations through the ABPN or the United Council for Neurologic Subspecialties.^28,29^ As such, there is a growing need for resources for trainee self-assessment and program evaluation. Our survey results suggest that in-training assessments are a resource that trainees and program directors find valuable for identifying areas of strength and opportunities for growth at both individual and programmatic levels. Subspecialty organizations are a natural source for the breadth and depth of expertise necessary for developing and maintaining high-quality in-training examinations. As we have shown with our experience, careful attention to specific components of quality and validity can help ensure that the in-training examination is optimal for the educational objectives. In Table 4, we summarize the valuable lessons we have learned over the first 3 years of EpiFITE implementation.

**Table 4:**
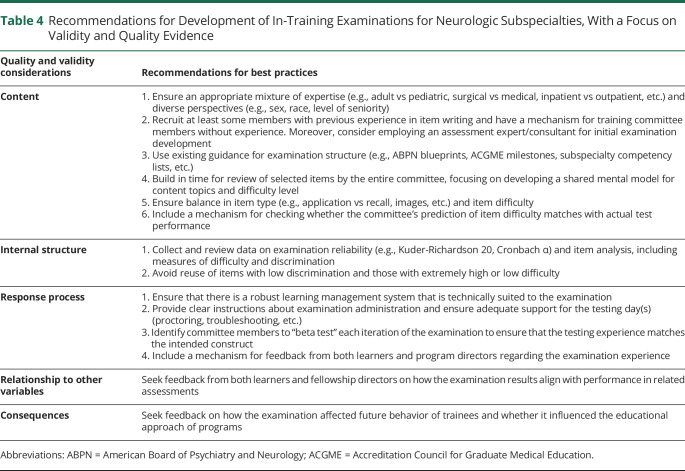
Recommendations for Development of In-Training Examinations for Neurologic Subspecialties, With a Focus on Validity and Quality Evidence

Quality and validity considerations	Recommendations for best practices
Content	1. Ensure an appropriate mixture of expertise (e.g., adult vs pediatric, surgical vs medical, inpatient vs outpatient, etc.) and diverse perspectives (e.g., sex, race, level of seniority) 2. Recruit at least some members with previous experience in item writing and have a mechanism for training committee members without experience. Moreover, consider employing an assessment expert/consultant for initial examination development 3. Use existing guidance for examination structure (e.g., ABPN blueprints, ACGME milestones, subspecialty competency lists, etc.) 4. Build in time for review of selected items by the entire committee, focusing on developing a shared mental model for content topics and difficulty level 5. Ensure balance in item type (e.g., application vs recall, images, etc.) and item difficulty 6. Include a mechanism for checking whether the committee's prediction of item difficulty matches with actual test performance
Internal structure	1. Collect and review data on examination reliability (e.g., Kuder-Richardson 20, Cronbach α) and item analysis, including measures of difficulty and discrimination 2. Avoid reuse of items with low discrimination and those with extremely high or low difficulty
Response process	1. Ensure that there is a robust learning management system that is technically suited to the examination 2. Provide clear instructions about examination administration and ensure adequate support for the testing day(s) (proctoring, troubleshooting, etc.) 3. Identify committee members to “beta test” each iteration of the examination to ensure that the testing experience matches the intended construct 4. Include a mechanism for feedback from both learners and program directors regarding the examination experience
Relationship to other variables	Seek feedback from both learners and fellowship directors on how the examination results align with performance in related assessments
Consequences	Seek feedback on how the examination affected future behavior of trainees and whether it influenced the educational approach of programs

Abbreviations: ABPN = American Board of Psychiatry and Neurology; ACGME = Accreditation Council for Graduate Medical Education.

Annual program evaluation has allowed us to identify the need for changes to our examination development process. Recently implemented changes include a system of mentorship of new committee members by more experienced members of the committee. This can allow each new committee member the opportunity for one-on-one review of items prior to full committee review, thus embedding an element of faculty development for each new committee member, ensuring that the content of the examination is of the highest quality. At the level of response process, we have implemented “beta testing” of the examination by at least 2 independent committee members to ensure that the items are accurate and provoke an appropriate response in test-takers. Future directions will include faculty development on the use of inclusive language in item writing and training on the recognition of bias in the development of high-stakes examinations (content) and incorporating item analysis into the committee review so that the entire group can engage in the evaluation of internal structure of the examination.

Our aim in sharing our experience in the development and evaluation of the EpiFITE is to ensure that other neurology subspecialty organizations can learn from our experiences as they consider the development of their own in-training examinations. Assessment can drive learning, and professional organizations have an opportunity to use best practices in examination development to promote the highest quality of neurologic care provided by each successive generation of practitioners.

## Appendix Authors

**Table:**

Name	Location	Contribution
Jeremy Moeller, MD, MSc	Department of Neurology, Yale School of Medicine, New Haven, CT	Drafting/revision of the article for content, including medical writing for content; major role in the acquisition of data; study concept or design; and analysis or interpretation of data
Ernesto Gonzalez-Giraldo, MD	Departments of Neurology and Pediatrics, University of California, San Francisco	Drafting/revision of the article for content, including medical writing for content; major role in the acquisition of data; study concept or design; and analysis or interpretation of data.
France W. Fung, MD, MSc	Departments of Neurology and Pediatrics, Children's Hospital of Philadelphia and University of Pennsylvania Perelman School of Medicine, Philadelphia	Drafting/revision of the article for content, including medical writing for content; major role in the acquisition of data
Emily L. Johnson, MD	Department of Neurology, Johns Hopkins School of Medicine, Baltimore, MD	Drafting/revision of the article for content, including medical writing for content; major role in the acquisition of data
Ammar Kheder, MD, MRCP	Department of Neurology and Pediatric Institute, Emory University School of Medicine, Atlanta, GA	Drafting/revision of the article for content, including medical writing for content; major role in the acquisition of data
Jane MacLean, MD, MS	Department of Pediatric Neurology, Sutter Medical Foundation, Mountain View, CA	Drafting/revision of the article for content, including medical writing for content; major role in the acquisition of data
Emily L. McGinnis, MD	Neurology Department, Kaiser Permanente Los Angeles Medical Center, CA	Drafting/revision of the article for content, including medical writing for content; major role in the acquisition of data
Wolfgang G. Muhlhofer, MD	University of Washington Regional Epilepsy Center, University of Washington, Seattle	Drafting/revision of the article for content, including medical writing for content; major role in the acquisition of data
Joel M. Oster, MD	Department of Neurology, Tufts University, Boston, MA	Drafting/revision of the article for content, including medical writing for content; major role in the acquisition of data
Sarah Schmitt, MD	Department of Neurology, Medical University of South Carolina, Charleston	Drafting/revision of the article for content, including medical writing for content; major role in the acquisition of data
P. Emanuela Voinescu, MD, PhD	Division of Epilepsy, Department of Neurology, Brigham and Womens Hospital, Harvard Medical School, Boston, MA	Drafting/revision of the article for content, including medical writing for content; major role in the acquisition of data
Lily C. Wong-Kisiel, MD	Department of Neurology, Mayo Clinic College of Medicine, Rochester, MN	Drafting/revision of the article for content, including medical writing for content; major role in the acquisition of data
Kandice J. Kidd, MA	American Epilepsy Society, Chicago, IL	Drafting/revision of the article for content, including medical writing for content; major role in the acquisition of data
Amy Kephart, MPH, CAE	American Epilepsy Society, Chicago, IL	Drafting/revision of the article for content, including medical writing for content; major role in the acquisition of data
Fred A. Lado, MD, PhD	Department of Neurology, Zucker School of Medicine at Hofstra-Northwell, Hempstead, NY	Drafting/revision of the article for content, including medical writing for content; major role in the acquisition of data
Sudha Kilaru Kessler, MD, MSCE	Departments of Neurology and Pediatrics, Children's Hospital of Philadelphia and University of Pennsylvania Perelman School of Medicine, Philadelphia	Drafting/revision of the article for content, including medical writing for content; major role in the acquisition of data; study concept or design; and analysis or interpretation of data

## Glossary

ABPN: American Board of Psychiatry and Neurology
ACGME: Accreditation Council for Graduate Medical Education
AES: American Epilepsy Society
COVID-19: coronavirus disease 2019
EpiFITE: Epilepsy Fellowship In-Training Examination
NS: nonsignificant
RITE: Residency In-Service Training Examination
